# Novel Sulfated Polysaccharides Disrupt Cathelicidins, Inhibit RAGE and Reduce Cutaneous Inflammation in a Mouse Model of Rosacea

**DOI:** 10.1371/journal.pone.0016658

**Published:** 2011-02-09

**Authors:** Jianxing Zhang, Xiaoyu Xu, Narayanam V. Rao, Brian Argyle, Lindsi McCoard, William J. Rusho, Thomas P. Kennedy, Glenn D. Prestwich, Gerald Krueger

**Affiliations:** 1 Center for Therapeutic Biomaterials, University of Utah, Salt Lake City, Utah, United States of America; 2 Department of Medicinal Chemistry, University of Utah, Salt Lake City, Utah, United States of America; 3 Department of Internal Medicine, University of Utah, Salt Lake City, Utah, United States of America; 4 Department of Pharmaceutics and Pharmaceutical Chemistry, University of Utah, Salt Lake City, Utah, United States of America; 5 Department of Dermatology, University of Utah, Salt Lake City, Utah, United States of America; The University of Queensland, Australia

## Abstract

**Background:**

Rosacea is a common disfiguring skin disease of primarily Caucasians characterized by central erythema of the face, with telangiectatic blood vessels, papules and pustules, and can produce skin thickening, especially on the nose of men, creating rhinophyma. Rosacea can also produce dry, itchy eyes with irritation of the lids, keratitis and corneal scarring. The cause of rosacea has been proposed as over-production of the cationic cathelicidin peptide LL-37.

**Methodology/Principal Findings:**

We tested a new class of non-anticoagulant sulfated anionic polysaccharides, semi-**s**ynthetic glycos**a**mino**g**lycan **e**thers (SAGEs) on key elements of the pathogenic pathway leading to rosacea. SAGEs were anti-inflammatory at ng/ml, including inhibition of polymorphonuclear leukocyte (PMN) proteases, P-selectin, and interaction of the **r**eceptor for **a**dvanced **g**lycation **e**nd-products (RAGE) with four representative ligands. SAGEs bound LL-37 and inhibited interleukin-8 production induced by LL-37 in cultured human keratinocytes. When mixed with LL-37 before injection, SAGEs prevented the erythema and PMN infiltration produced by direct intradermal injection of LL-37 into mouse skin. Topical application of a 1% (w/w) SAGE emollient to overlying injected skin also reduced erythema and PMN infiltration from intradermal LL-37.

**Conclusions:**

Anionic polysaccharides, exemplified by SAGEs, offer potential as novel mechanism-based therapies for rosacea and by extension other LL-37-mediated and RAGE-ligand driven skin diseases.

## Introduction

Rosacea is a common skin disease afflicting primarily Caucasian women of Celtic descent [1]. Rosacea is characterized by central erythema of the face, with telangiectatic blood vessels, papules and pustules, and can produce skin thickening, especially on the nose of men, creating rhinophyma. Rosacea can also produce dry, itchy eyes with irritation of the lids, keratitis and corneal scarring. The disease disfigures in a prominent manner, and its treatment is empiric and imperfect [2]. The pathogenesis of rosacea has been attributed in part to cutaneous over-production of a cationic anti-microbial cathelicidin peptide produced by the processing serine proteinase stratum corneum tryptic enzyme (SCTE) [3], [4]. Cathelicidins are highly cationic 18 kDa propeptides cleaved to an active 37-amino acid C-terminal anti-microbial peptide, LL-37 [5]. LL-37 induces interleukin-8 (IL-8) secretion by human keratinocytes, and injection of LL-37 into mouse skin recapitulates rosacea-like redness and PMN infiltration [3].

We have evaluated a family of sulfated and metabolically stabilized anionic polysaccharide derivatives known as semi-**s**ynthetic glycos**a**mino**g**lycan **e**thers (SAGEs). We hypothesized that a topically-applied SAGE could be used as a novel therapy for rosacea by binding and inhibiting the inflammatory activity of excess cationic cathelicidins. We show that one SAGE, GM-1111, exhibits substantial anti-inflammatory activities at nanomolar concentrations, including inhibition of cationic PMN proteases, inhibition of the leukocyte adhesion receptor P-selectin, and inhibition of the interaction of the **r**eceptor for **a**dvanced **g**lycation **e**nd-products (RAGE) with its disparate ligands. GM-1111 avidly bound LL-37 and inhibited IL-8 secretion in cultured human keratinocytes in response to LL-37 stimulation. When mixed with LL-37, SAGEs prevented the extensive erythema and PMN infiltration produced by direct intradermal injection of LL-37 into mouse skin [3]. More importantly, topical application of a 1% SAGE-containing emollient to overlying injected skin also substantially reduced the redness and cutaneous PMN infiltration induced by intradermal LL-37. Herein, data demonstrate anionic polysaccharides, exemplified by SAGEs, as the first mechanism-based therapy that targets the proposed molecular etiology of rosacea.

## Results

### SAGEs are non-animal derived

**Figure 1:**
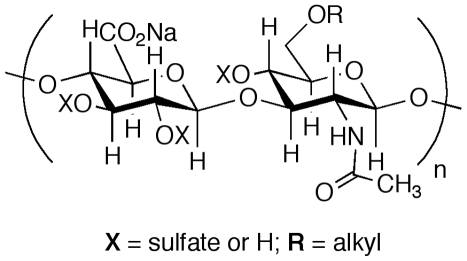
Structure of semi-synthetic glycosaminoglycan ethers (SAGEs). SAGEs can vary in molecular size, and in extent of alkylation and sulfation. GM-1111 is a low-molecular weight SAGE with an average molecular weight of 5.5 kDa.

Twenty-five novel derivatives of hyaluronic acid (HA) were obtained from GlycoMira, LLC (Salt Lake City, UT). HA is an immunoneutral skin polysaccharide consisting of long polymers (up to 10 MDa) of the disaccharide N-acetylglucosamine (GlcNAc) and glucuronic acid (GlcA) linked GlcNacβ1-3GlcAβ1-4 in repeating units along the chain. Fermentation-derived HA was chemically alkylated to provide lipophilicity to both improve dermal penetration and reduce hydrolysis by hyaluronidases [6]. Subsequently, the HA ethers were sulfated to adjust polyanionic charge and anti-inflammatory properties. The HA used as a starting material varied from 50 kDa to 950 kDa. A representative SAGE structure is illustrated in Figure 1. For further study, we chose the SAGE GM-1111, which was produced from 53 kDa HA and had a final molecular weight of 5.5 kDa.

### SAGEs bind P-selectin, Mac-1 and RAGE, and potently inhibit P-selectin, cationic PMN proteases and interaction of RAGE with its disparate ligands

The SAGE GM-1111 showed anti-inflammatory activities similar to those of heparin or its low anticoagulant analogs [6] in a number of *in vitro* assays.

**Figure 2:**
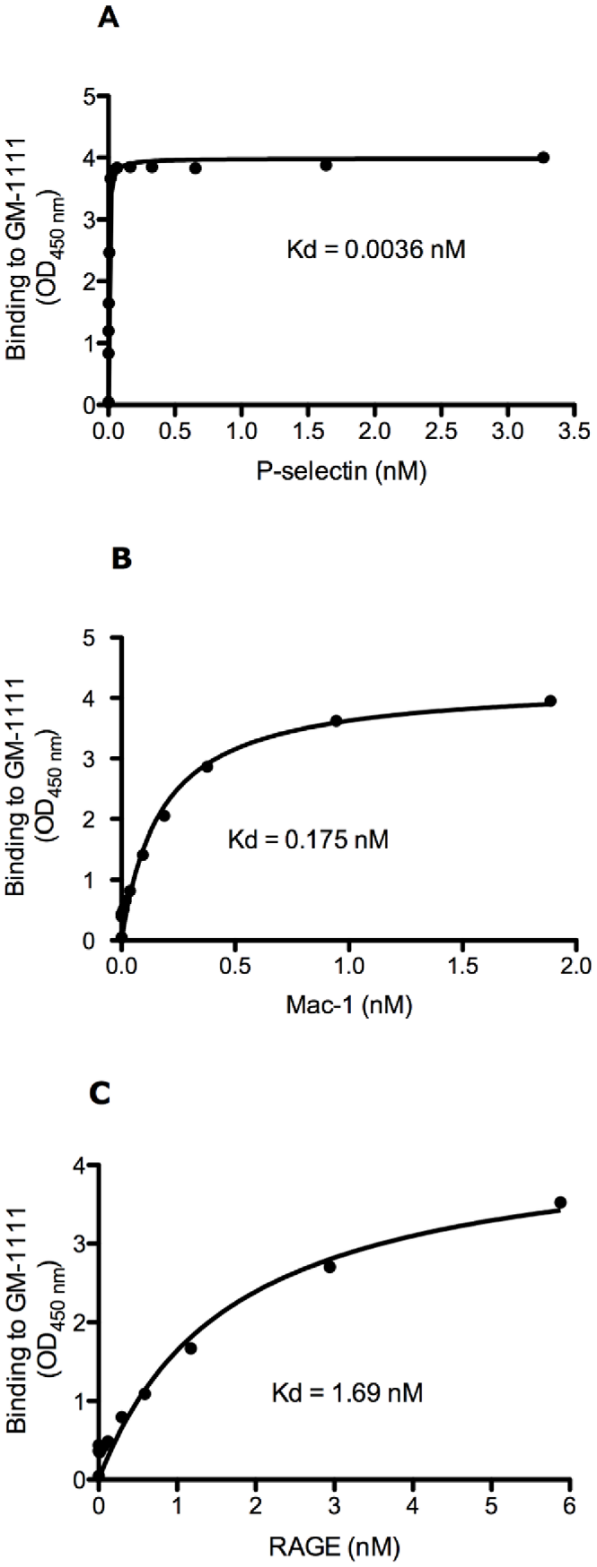
SAGEs bind to vascular adhesion proteins. GM-1111 was studied to determine binding affinity for P-selectin (**A**), Mac-1 (**B**), and RAGE (**C**). Binding affinity (K_D_) values were 0.0036 nM for GM-1111 binding to P-selectin, 0.175 nM for GM-1111 binding to Mac-1 and 1.69 nM for GM-1111 binding to RAGE.

First, SAGEs avidly bound to the adhesion molecule P-selectin, the Mac-1 integrin (CD11b/CD18) and the multi-ligand immunoglobulin superfamily receptor RAGE. Figure 2 shows that GM-1111 exhibited saturable binding to P-selectin with a K_D_ of 0.0036 nM (Figure 2A), to Mac-1 with a K_D_ of 0.175 nM (Figure 2B) and to RAGE with a K_D_ of 1.69 nM (Figure 2C).

**Figure 3:**
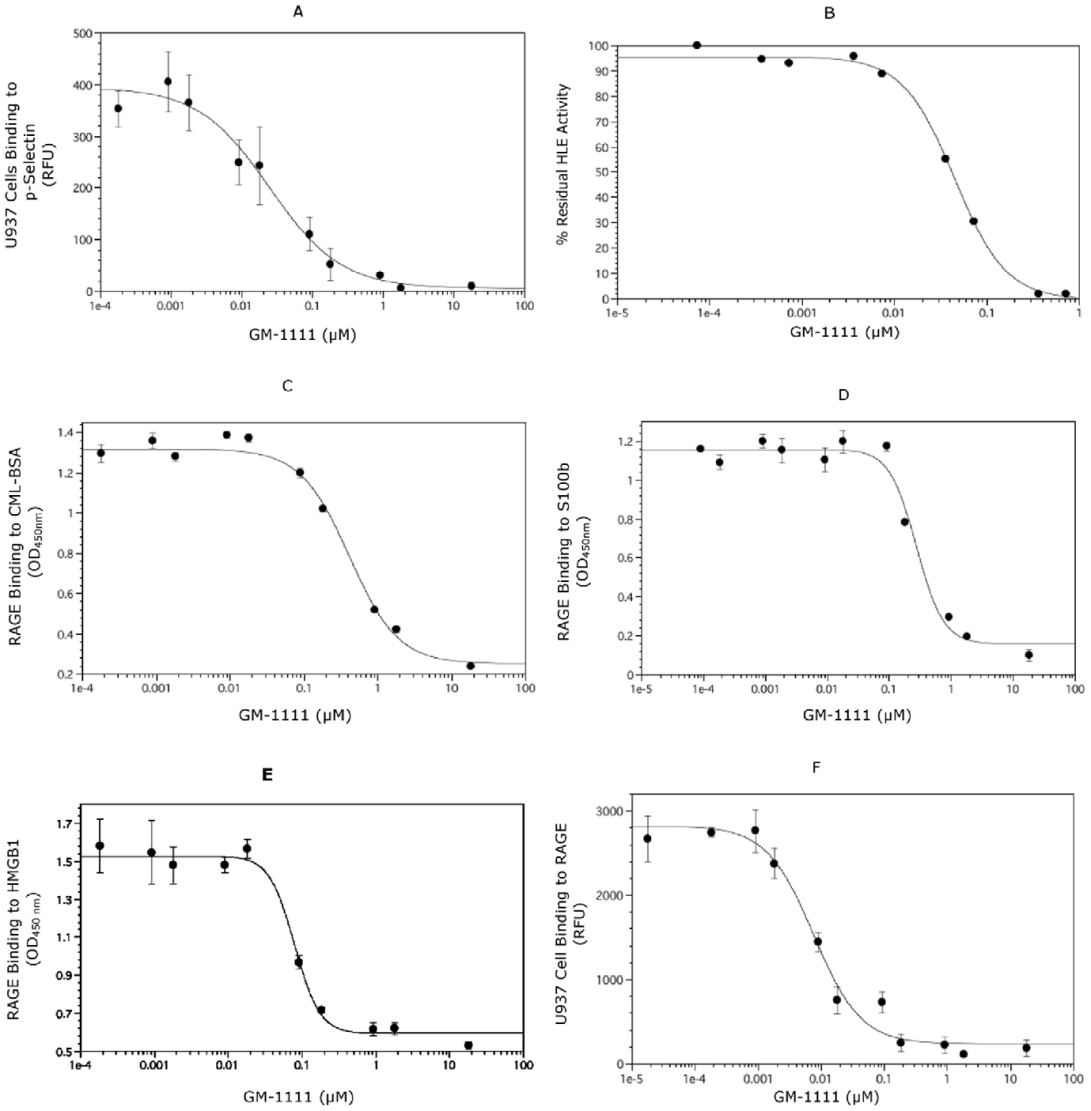
SAGEs inhibit P-selectin, HLE, interaction of RAGE with its many ligands. Data points in each figure represent the average value ± standard deviation of quadruplicate wells for each concentration of SAGE. **A.** SAGEs inhibit P-selectin. Inhibition of P-selectin glycoprotein ligand-1 (PSGL-1) binding to P-selectin by SAGEs was studied using calcein-labeled U937 cells incubated in microwells coated with P-selectin. After 1 h, plates were washed, bound cells were lysed with Triton-X100 buffer and bound cells were quantified using an excitation of 494 nm and emission of 517 nm. GM-1111 inhibits PSGL-1 attachment to P-selectin with an IC_50_ of 25 nM. **B.** SAGEs inhibit HLE. HLE (100 nM) was incubated with GM-1111 at 1–100 nm concentrations in 0.5 M HEPES buffer for 15 min. Following incubation, the elastase substrate, Suc-Ala-Ala-Val-*p*NA was added to the reaction mixture to the final concentration of 0.3 mM. Absorbance due to *p*-NA hydrolysis was monitored for 15 min at absorbance of 405 nm. GM-1111 inhibits HLE with an IC_50_ of 45 nM. **C–E.** SAGEs inhibit interaction of the AGE product CML-BSA, the calgranulin S100b and the alarmin HMGB-1 with RAGE. Microwell plates coated with CML-BSA (**C**), S100b calgranulin (**D**) or HMGB-1 (**E**) were incubated with RAGE-Fc chimera with or without GM-1111 for 2 h. Plates were washed, incubated with anti-RAGE antibody, incubated for 1 h, washed again four times and incubated with horse-radish peroxidase conjugated secondary for 1 h. A colorimetric reaction was produced by addition of tetramethyl benzidine chromogen (TMB) and quantified by absorbance at 450 nm. GM-1111 inhibits interaction of RAGE with CM-BSA, S100b and HMGB-1 with IC_50_ values of 413, 275 and 80 nM, respectively. **F.** SAGEs inhibit function of RAGE as an adhesion ligand. Inhibition of Mac-1-dependent ligation of RAGE by U937 cells was studied as in **A**, but using microwells coated with RAGE. GM-1111 inhibits Mac-1 attachment to RAGE with an IC_50_ of 7.6 nM.

Second, SAGEs were potent inhibitors of the leukocyte adhesion molecule P-selectin [7]. Competitor-mediated displacement of U937 human monocytes, which loosely adhere to P-selectin through P-selectin glycoprotein ligand-1 (PSGL-1), was studied using fluorescent-labeled U937 cells. Table 1 and Figure 3A show that the SAGE GM-1111 inhibited U937 binding to P-selectin with a 50% inhibitory concentration (IC_50_) of 24.9 nM, approximately three times higher than the IC_50_ for heparin (7.9 nM).

**Table 1:** Anti-Inflammatory Activities of SAGE, GM-1111, *In Vitro* 1.

Anti-Inflammatory Assay	IC_50_ values (nM)	
	SAGE*	Heparin*
1U937 monocyte binding to P-selectin	24.9	7.9
2Human Leukocyte Elastase activity	44.7	14.9
3CML-BSA binding to RAGE	412.7	27.8
3S100b binding to RAGE	274.5	92.1
3HMGB-1 binding to RAGE	79.6	2.9
1U937 monocyte binding to RAGE	7.6	7.9

*The average molecular mass of SAGE is 5.5 kDa and heparin is 14 kDa.

Details of ^1^cell surface binding assays, ^2^ inhibition of human leukocyte elastase (HLE) and ^3^solid phase binding assays are found in Methods. Detailed graphical plots of the results from cell surface binding assays, inhibition of HLE and solid phase binding assays are shown in Figure 3.

Third, as sulfated polyanions, SAGEs were potent inhibitors of cationic PMN proteases such as human leukocyte elastase [8]. GM-1111 inhibited the PMN protease human leukocyte elastase (HLE) with an IC_50_ of 44.7 nM (Table 1 and Figure 3B), approximately three times higher than the IC_50_ of 14.9 nM for heparin. Thus, the polyanionic nature of the SAGEs would be expected to reduce protease activity in part *via* electrostatic interactions. However, this simplistic explanation cannot account for the totality of observed SAGE pharmacology *in vitro* [6].

Fourth, SAGEs were found to be potent inhibitors of RAGE binding with its ligands. The advanced glycation end-product (AGE) carboxymethyl lysine-modified (CML) protein is prominently formed in the dermis as the consequence of sun exposure, and avidly ligates and activates RAGE inflammatory signaling [9]. RAGE binds to immobilized CML-bovine serum albumin (BSA) in a dose dependent manner with an equilibrium constant (K_D_) of 0.43 nM [10]. GM-1111 potently inhibited interaction of CML-BSA with RAGE (Table 1 and Figure 3C) with an with an IC_50_ of 413 nM. Once PMNs have migrated into inflamed dermis, PMN secretion of S100 calgranulins provides an active RAGE ligand to perpetuate inflammatory signaling even in the absence of AGE products [11]. RAGE engages immobilized S100b in a dose-dependent manner with a K_D_ of 0.45 nM [10]. GM-1111 inhibited ligation of RAGE by S100b calgranulin with an IC_50_ of 275 nM, compared with IC_50_ of 92 nM for heparin (Table 1 and Figure 3D) Likewise, RAGE binds immobilized human high mobility box group protein-1 (HMGB-1) in a saturable fashion with a K_D_ of 0.64 nM [10]. HMGB-1 is secreted by monocytes and macrophages as an inflammation producing cytokine and is also released by necrotic cells into areas of injury [12]. GM-1111 inhibited ligation of RAGE by HMGB-1 with an IC_50_ of 80 nM, compared with an IC_50_ of 2.9 nM for heparin (Table 1 and Figure 3E). Finally, using the Mac-1 (CD11b/CD18) as a counter-ligand, leukocytes ligate RAGE on vascular endothelium as an adhesion molecule essential for exiting the circulation into areas of inflammation [13]. GM-1111 inhibited binding of U937 monocytes to RAGE with an IC_50_ of 7.6 nM, essentially equipotent with the IC_50_ of 7.9 nM for heparin (Table 1 and Figure 3F). Taken together, these results suggest that SAGEs may provide considerable anti-inflammatory activity in diseases mediated by RAGE-ligand interactions, including the skin, where RAGE likely plays a role in lymphocyte-mediated diseases such as atopic dermatitis and psoriasis, as well as photo-ageing.

### SAGEs are safe when administered topically

When applied to cultured human dermal fibroblasts (nHDF) and keratinocytes (nHEK), GM-1111 did not inhibit proliferation or cause cell toxicity up to 1 mg/ml (data not shown). Furthermore, none of the SAGEs tested, including GM-1111, elicited any skin reaction in Draize tests up to 10 mg/ml [14] (data not shown). SAGEs are also effectively non-anticoagulant in nature; for example, GM-1111 has approximately 0.2–0.5% of the anticoagulant activity of heparin. Specifically GM-1111 shows values of 0.3 IU/mg anti-Xa activity and 0.8 IU/mg anti-IIa activity, compared to 150–160 IU/mg each for unfractionated heparin. Unlike heparin, many highly charged polyanionic polymers are potent activators of Factor XII (Hagemann factor), secondarily producing kinins [15]. GM-1111 failed to show any activation of Factor XII [16], even at concentrations 10 to 100-fold higher than needed to achieve pharmacologic inhibition of inflammation (data not shown). From these data, we concluded that the GM-1111 was safe as a lead candidate for topical use.

### SAGEs bind to LL-37 and inhibit the biologic activity of LL-37 *in vitro*

**Figure 4:**
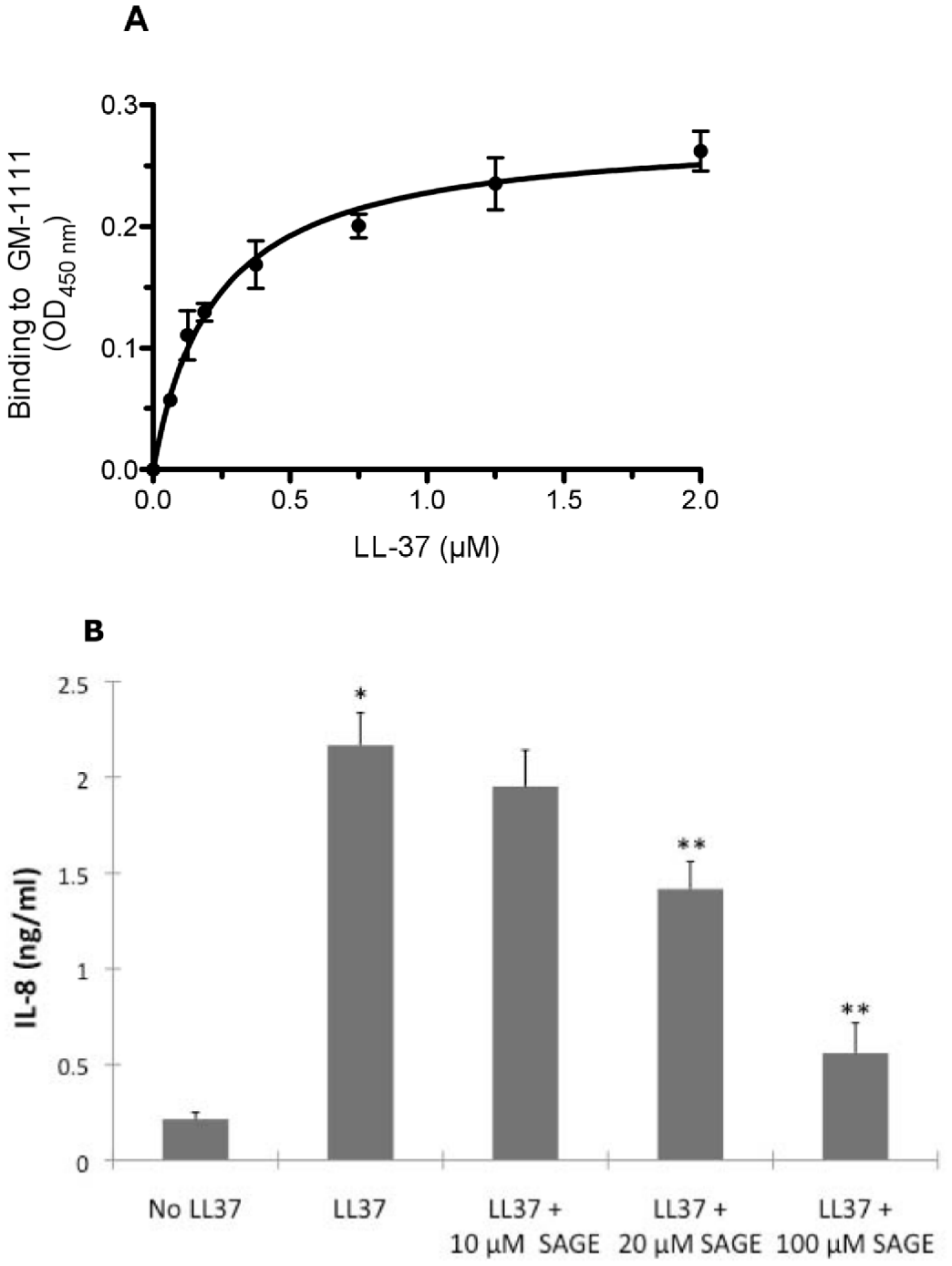
SAGE binds LL-37 and inhibits LL-37-induced interleukin-8 (IL-8) expression *in vitro.* **A.** SAGE binds LL-37. Microwell plates coated with GM-111 were incubated with LL-37 at 37°C for 2 h. Plates were washed, incubated with anti-LL-37 antibody, incubated for 1 h, washed again four times and incubated with peroxidase-conjugated secondary antibody for 1 h. A colorimetric reaction was produced by addition of TMB and quantified by absorbance at 450 nm. LL-37 binds to GM-1111 with a K_D_ of 0.225 nM. **B.** SAGE inhibits IL-8 production LL-37 stimulated keratinocytes. Human keratinocytes were grown to confluence and treated with 3.2 µM LL-37 or LL-37 and a 4x molar excess of GM-111101 for 6 h. Supernatants were collected and placed in a sterile 96-well plate. Production of IL-8 was determined by ELISA (R&D Systems, Minneapolis, MN) in accordance with manufacturer's instructions. Co-addition of GM-1111 significantly inhibited IL-8 release into medium. *P<0.01 as compared with negative control group without LL37; **P<0.05 as compared with positive control group without GM-111101 treatment.

The SAGE GM-1111 bound to the polycationic LL-37 peptide in a saturable fashion with a K_D_ of 0.225 nM (Figure 4A). This suggests that at least some of the biologic activity of the polyanionic SAGEs could be due to saturable, charge-neutralization of the cationic cathelicidins. However, electrostatic interactions alone cannot account for the overall SAGE pharmacology. For example, similar to previous reports [3], LL-37 induced IL-8 secretion by cultured human keratinocytes (Figure 4B). Addition of GM-1111 to the medium in addition to LL-37 significantly reduced IL-8 production by cultured keratinocytes (Figure 4B).

### SAGEs inhibit cutaneous inflammation from intradermal LL-37

**Figure 5:**
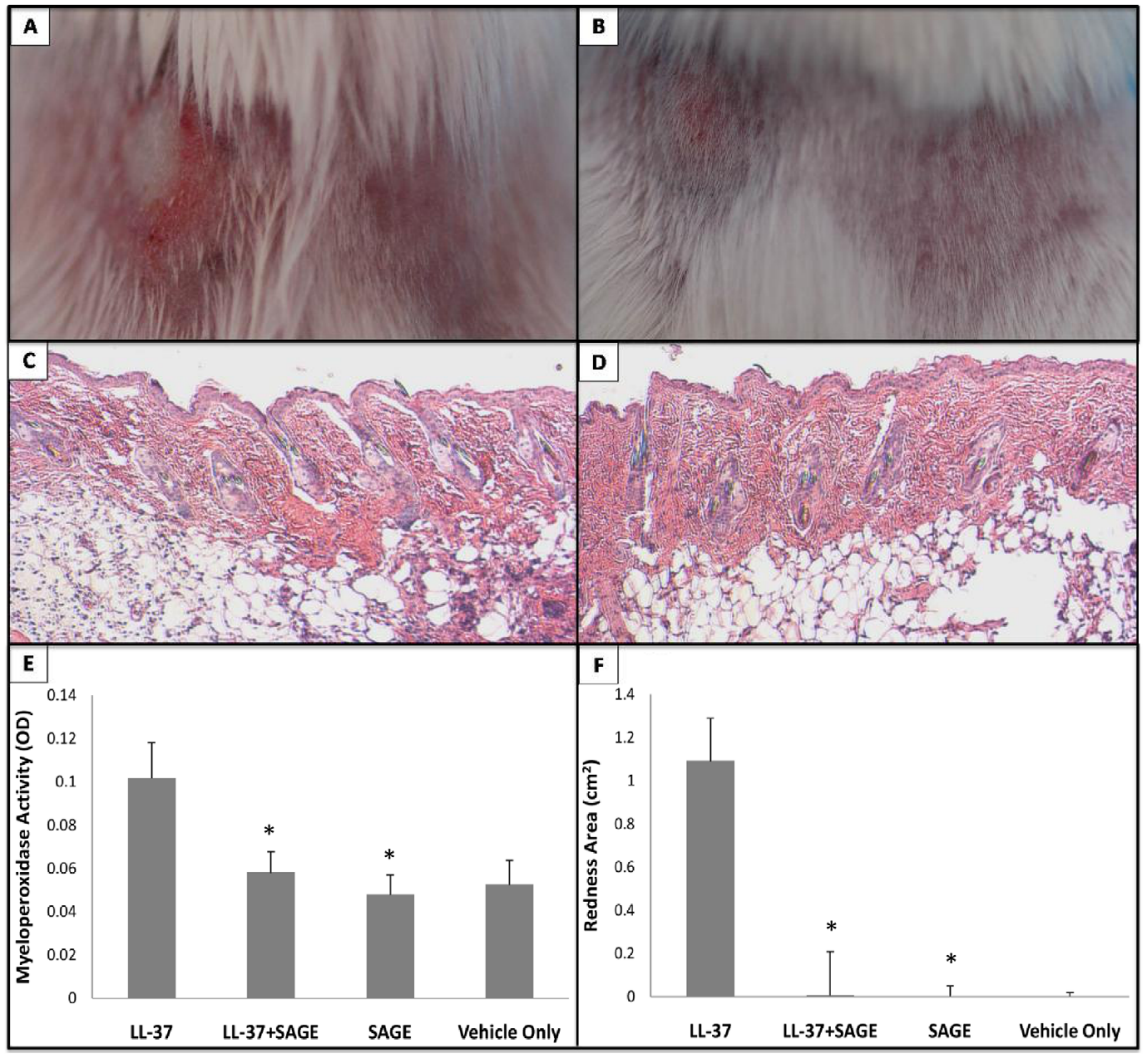
SAGE co-injection inhibits erythema and PMN infiltration from intradermal LL-37. Balb/c mice were shaved to expose an area of skin on the back. Twenty-four hours later, mice were intradermally injected with 40 µl of vehicle (nanopure water), LL-37 (at 320 µM concentration in water), SAGE GM-1111 (1,280 µM in water), or LL-37+ GM-1111 mix (peptide +4 molar equivalents of SAGE) placed intradermally into the shaved skin using a 31-gauge needle and 0.5 ml insulin syringe. Injections were repeated every 12 h thereafter. Forty-eight hours after the initial injection (four injections in total), animals were lightly anesthetized with isoflurane, the area of injected skin was photographed, the intensity of erythema was assessed as a redness score (from 1 to 5, with 5 as the most red), and the area of erythema was measured with micrometer calipers. The injected skin was then biopsied for histopathologic staining and to assess PMN infiltration through measurement of myeloperoxidase (MPO) activity. **A**. Gross picture of LL-37 injected skin region. **B**. Co-injection model of LL-37 and SAGE GM-1111. **C**. H&E-stained cross-sectional view of a LL-37 injected skin sample. **D**. H&E-stained cross-sectional view of a LL-37 mixed with GM-1111- injected skin region. **E**. MPO activity measurement of LL-37 injection with different treatments. **F**. Area of erythema from LL-37 injection. *P<0.05 vs intradermal LL-37 alone; n = 6 per group.

**Figure 6:**
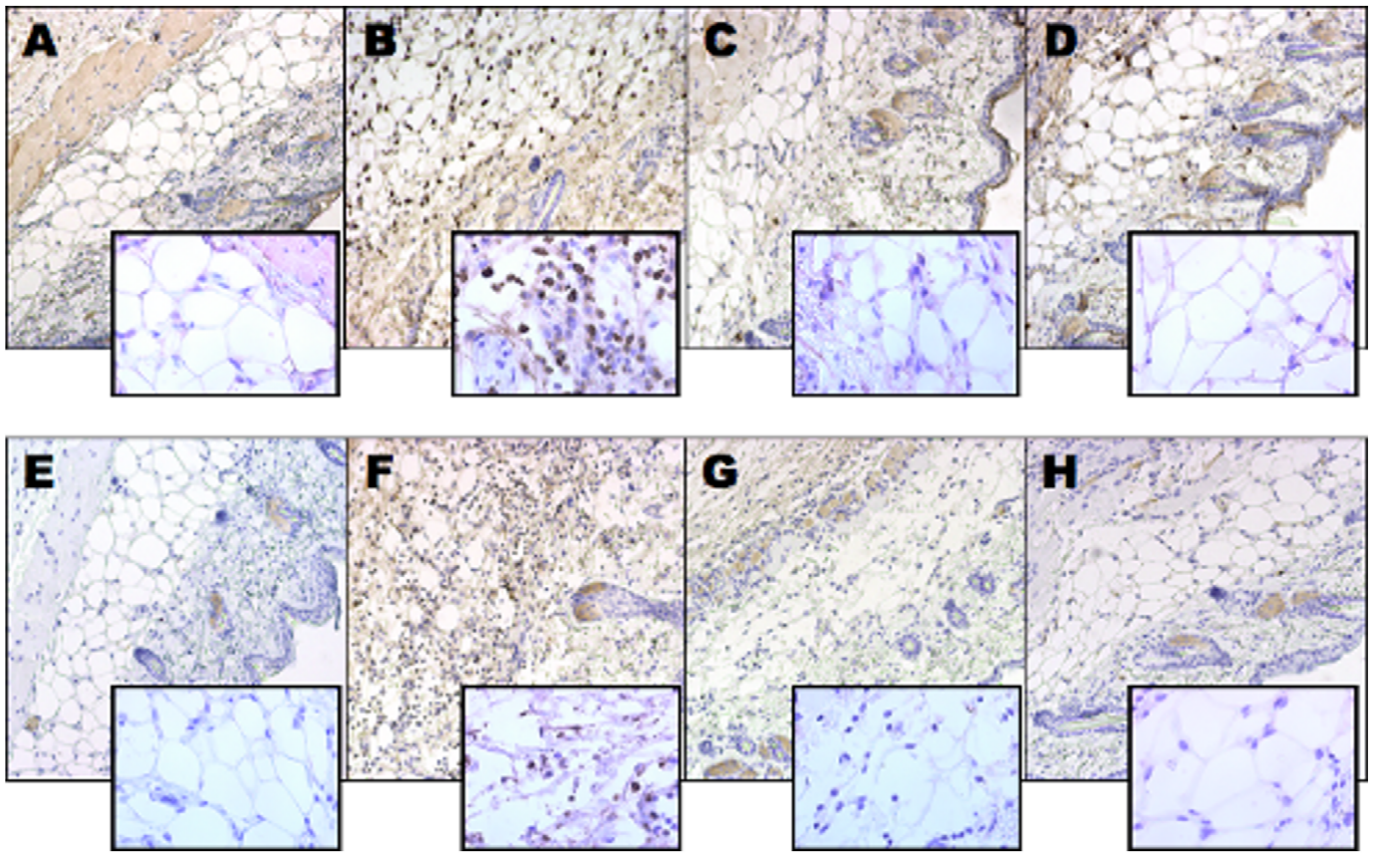
Topical SAGE reduces PMN infiltration and S100 calgranulin accumulation from intradermal LL-37. LL-37, PBS and LL-37 and SAGE-injected mouse skin was stained with antibody to mouse Gr-1 to examine neutrophil infiltration, and also for S100A8 calgranulin, the major RAGE-binding calgranulin present in murine PMNs [17] as described in Methods. **A.** Staining of normal control skin for the PMN antigen Gr-1. **B.** Staining of LL-37 injected skin for the PMN antigen Gr-1. **C.** Staining of skin injected with LL-37 plus GM-1111 (applied at t = 0) for the PMN antigen Gr-1. **D.** Staining of skin injected with LL-37 plus GM-1111 (applied at t = 12 h) for the PMN antigen Gr-1. **E.** Staining of normal control skin for the RAGE ligand S100A8. **F.** Staining of LL-37 injected skin for the RAGE ligand S100A8. **G.** Staining of skin injected with LL-37 plus GM-1111 (applied at t = 0) for the RAGE ligand S100A8. **H.** Staining of skin injected with LL-37 plus GM-1111 (applied at t = 12 h) for the RAGE ligand S100A8. Figures and insets are shown at 20x and 40x magnification, respectively.

Intradermal injection of LL-37 into Balb/c mice at four 12-h intervals for 48 h produced cutaneous erythema with central necrosis (Figure 5A), prominent intradermal PMN infiltration (Figure 5C and Figure 6B), marked edema of the dermis (Figure 5C) and increased tissue myeloperoxidase (MPO) activity (Figure 5E). This reproduced the previously described murine model of rosacea [3]. The area of erythema (Figures 5B and F) and redness score (3.8±0.5 after LL-37 alone vs 1.2±0.5 after LL-37+ SAGE, P<0.05) were both significantly reduced by co-injection of GM-1111 with LL-37. Simultaneous injection of GM-1111 with LL-37 also significantly decreased PMN infiltration, as assessed by histology (Figure 5D) or tissue MPO activity (Figure 5E). Thus, at a minimum, charge neutralization of LL-37 by SAGE co-injection results in a significant reduction of LL-37-induced inflammation.

**Figure 7:**
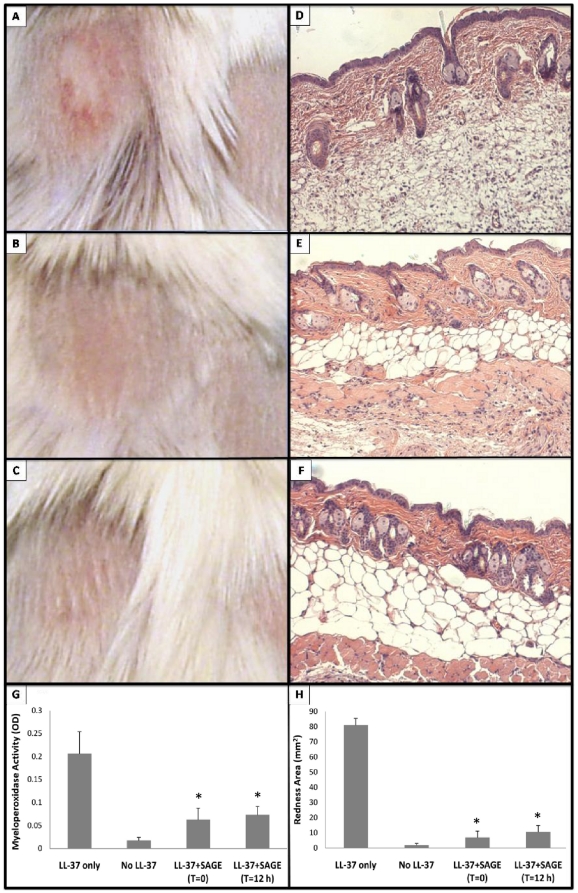
Topical SAGE reduces LL-37-induced inflammation. Balb/c mice were injected intradermally as described in Figure 5, and treated topically with GM-1111-1 (1% w/w) in a triglyceride-based emollient. **A–C**. Gross pictures of LL-37 injected skin region: **A**, no treatment; **B**, GM-1111 treatment immediately (t = 0) after LL-37 injection; **C**, GM-1111 treatment beginning at t = 12 h after LL-37 injection. **D**. H&E-stained cross-sectional view of a LL-37 injected skin sample. **E**. H&E-stained cross-sectional view of GM-1111 treatment at t = 0 in LL-37 injected skin region. **F**. H&E-stained cross-sectional view of GM-1111 at t = 12 h treatment in LL-37 injected skin region. **G**. MPO activity measurement of LL-37 injection with different treatments. **H**. Area of erythema from LL-37 injection. *P<0.05 vs intradermal LL-37 alone; n = 6 per group.

**Figure 8:**
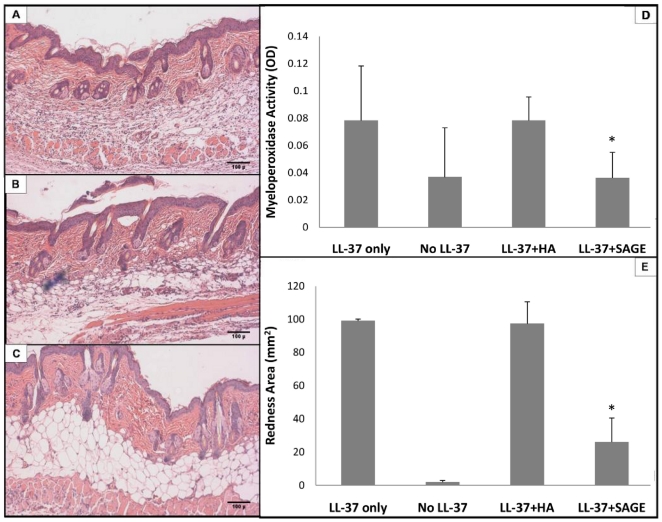
HA emollient does not reduce LL-37-induced inflammation. Experiments were performed as in Figure 7, but with immediate (t = 0) application of 1% w/w HA-containing emollient (53 kDa HA) or 1% w/w GM-1111 emollient. *P<0.05 vs LL-37 alone. **A**. H&E-stained cross-sectional view of LL-37 injected skin. **B**. H&E-stained section of LL-37 injected skin treated with topical HA emollient alone. **C**. H&E-stained section of LL-37 injected skin treated with topical emollient containing 1% SAGE GM-1111. **D**. MPO activity measurement of LL-37 injection with different treatments. **E**. Area of erythema from LL-37 injection. Erythema scores after LL-37 injection were: 5.1±0.1 after LL-37 alone; 4.9±0.44 after LL-37+ HA; 2.3±0.7 after LL-37+ SAGE (P<0.05 LL-37 alone vs LL-37+ SAGE). *P<0.05 vs intradermal LL-37 alone; n = 6 per group.

**Figure 9:**
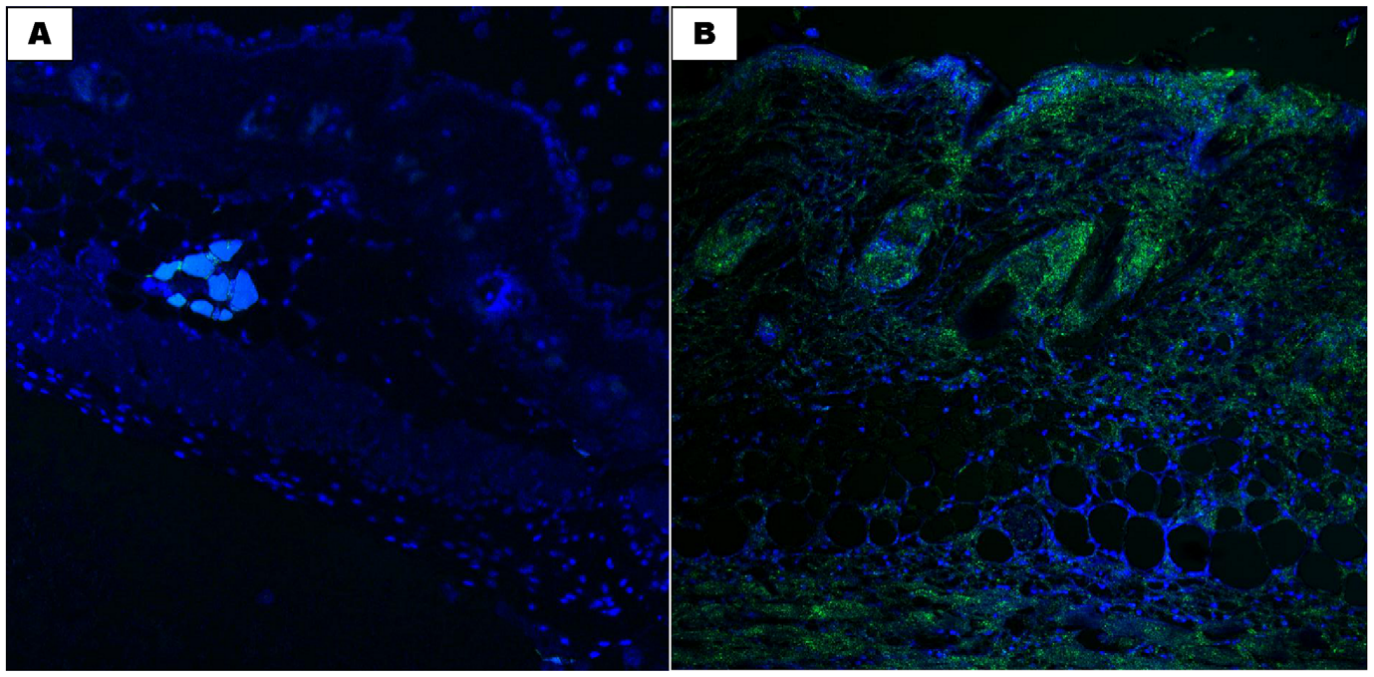
Topical SAGE penetrates into inflamed mouse skin. Balb/c mice were injected intradermally with LL-37 every 12 h for 48 h as in Figure 7. Control emollient [3% (w/w) methylcellulose] or emollient containing Alexa Fluor 633-labeled GM-1111 was applied topically to the injected skin after the last two LL-37 injections. Under low light conditions, skin was harvested 12 h after the last emollient application, fixed in paraformaldehyde and embedded in paraffin. Slides were deparaffinized with xylene, stained with DAPI (1∶100 dilution in fluorescent mounting medium) and imaged using an FV1000 confocal microscope. The skin surface is oriented at the top of each image. **A**. Skin from controls demonstrates blue DAPI (λ_ex_ = 358 nm; λ_em_ = 461 nm) image but no SAGE fluorescence. **B.** Skin treated with Alexa Fluor 633-labeled GM-1111 demonstrates fluorescence (λ_ex_ = 633 nm; λ_em_ = 647 nm; false colored green in the image) consistent with SAGE penetration into LL-37-inflamed skin.

To determine if topical SAGEs were effective topical anti-inflammatory agents in this rosacea model, we formulated GM-1111 into a standard triglyceride-based transdermal emollient that contained 20% (w/w) water and 1% (w/w) of active drug. LL-37 was injected intradermally every 12 h for 48 h. In treated animals (n = 6 per group), the injected skin was gently rubbed with drug-free emollient (control) or emollient with 1% GM-1111 either immediately (t = 0) or 12 h after the first injection (t = 12 h). Emollient application was repeated after each of the intradermal LL-37 injections. Cutaneous inflammation from intradermal LL-37 was significantly reduced by topical 1% GM-1111 (Figure 7). Whether applied at t = 0 or t = 12 h after the first LL-37 injection, topical GM-1111-containing emollient significantly reduced both erythema (t = 0, Figure 7B; t = 12 h, Figure 7C) and PMN infiltration (t = 0, Figures 7E, 7G and Figure 6C; t = 12 h, Figures 7F, 7G and Figure 6D) relative to untreated or vehicle only animals receiving the intradermal LL-37. The SAGE-containing emollient also significantly reduced the area of erythema (Figure 7H) and redness score [4.6±0.3 after LL-37 alone, 1.6±0.4 after LL-37+ SAGE (t = 0) and 1.5±0.5 after LL-37+ SAGE (t = 12 h), both P<0.05 vs LL-37 alone], and decreased MPO activity within biopsies of injected skin (Figure 7G). Anti-inflammatory effects were not evident using 1% concentration of 53 kDa HA in the emollient in place of the GM-1111 (Figure 8). Confocal microscopy was performed on LL-37 injected mouse skin after topical treatment with emollient containing fluorescent-labeled GM-1111 (Figure 9). In the presence of irritant disruption of the stratum corneum, labeled SAGE penetrated deeply into inflamed skin. These results support our hypothesis that the SAGE GM-1111, but not HA, would be topically effective in a pharmaceutically acceptable emollient to treat the cathelicidin peptide-mediated elements of inflammation associated with rosacea.

S100 calgranulins have not been previously reported as pathobiologically important in rosacea, but their presence would not be surprising in light of the prominent PMN infiltration in this disease [1]. Immunohistochemical staining for the RAGE ligand S100A8 confirmed the presence of this mouse homologue for the human leukocyte S100 calgranulin S100A12 [17] in LL-37-injected skin (Figures 6E vs 6F). Importantly, topical GM-1111 treatment reduced S100A8 staining expression compared to LL-37 alone (Figures 6G and 6H vs 6F).

### SAGEs inhibit cutaneous inflammation from croton oil

**Figure 10:**
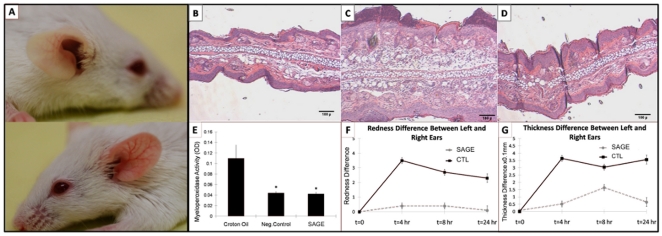
Topical SAGE reduces ear edema from croton oil. GM-1111 (1%) was applied topically in the croton oil model. **A**. Normal control right ear (top) and croton oil painted left ear (bottom). **B**. H&E section of control normal ear. **C**. H&E-stained cross-sectional view of a croton oil painted ear with vehicle treatment. **D**. H&E-stained cross-sectional view of croton oil painted ear with SAGE treatment. **E**. MPO activity measurement in ears. **F**. Ear thickness differences between left (croton oil) and right (normal) ears. **G**. Redness difference between right and left ears. *P<0.05 vs croton oil with vehicle emollient alone (negative control).

As another model, we employed croton oil, which is commonly used to study PMN-mediated skin inflammation in screening anti-inflammatory compounds for dermatologic use [18]. Croton oil contains the phorbol ester tetradecyl phorbol acetate (TPA), which activates protein kinase C in skin cells, producing abundant chemotaxins which can signal PMN influx. Cutaneous inflammation from croton oil has also recently been linked in part to recruitment of inflammatory cells by HMGB-1 released from TPA-exposed keratinocytes [19]. When painted on the ear, croton oil produces intense redness (Figure 10A bottom), accompanied by edema (Figure 10C) and intradermal increase in MPO activity (Figure 10E), indicative of PMN infiltration, compared to the control ear (Figures 10A top, B and E). Topical application of GM-1111 significantly decreased phorbol-induced ear edema (Figure 10D), MPO activity (Figure 10E), increase in erythema (Figure 10F), and increase in ear thickness (Figure 10G). This indicates that topical SAGEs may have utility in treating other dermatoses that are in part or completely mediated by PMNs.

## Discussion

Herein we show that novel sulfated glycosaminoglycan ethers have broad anti-inflammatory activity. The SAGE GM-1111 avidly bound to the leukocyte adhesion molecule P-selectin, the Mac-1 integrin and the pro-inflammatory pattern recognition receptor RAGE. This SAGE also potently inhibited P-selectin, blocked catalytic activity of the cationic PMN protease HLE, and inhibited the interaction of RAGE with its disparate ligands, including the AGE product CML-BSA, the S100 calgranulin S100b and the nuclear alarmin HMGB-1. Furthermore, GM-1111 demonstrated the ability to block leukocytes from using RAGE as an alternative adhesion ligand. The activities exemplified by GM-1111 (Table 1 and Figures 2 and 3) are generally demonstrated by a wide range of SAGEs with distinct structures and distinctive structure-activity profiles [6]. In aggregate, these activities would be predicted to be broadly anti-inflammatory. We confirmed this to be the case using a recently reported model of human rosacea [3]. LL-37 avidly bound to SAGE (Figure 4A) and addition of GM-1111 to medium blocked the ability of LL-37 to induce IL-8 expression by cultured human keratinocytes (Figure 4B). Further, co-injection of GM-1111 with LL-37 inhibited development of erythema (Figures 5A vs B and F) and skin infiltration of PMNs (Figure 5C vs 5D and 5E) in response to intradermal LL-37 peptide. Topical GM-1111 was also effective at ameliorating LL-37 induced skin inflammation (Figure 7), and similarly reduced inflammatory edema and PMN infiltration in response to dermal application of croton oil (Figure 10). Thus, topical SAGEs are the first mechanistically based approach for treating rosacea that intersects with currently understood pathogenic mechanisms in this disease [3], and may also be therapeutic for other leukocyte mediated skin disorders.

Cathelicidin peptides are polycationic antimicrobial peptides important for innate immunity of skin and other organs [5]. In humans, the major cathelicidin peptide is LL-37, which is produced by proteolytic processing of its precursor hCAP18 (human cationic antimicrobial protein 18). In addition to being antimicrobial, antifungal and antiviral, LL-37 is chemoattractant through activation of the formyl peptide receptor-like 1 (FPRL1) receptor on leukocytes, and can induce keratinocyte IL-8 and IL-18 secretion through activation of the epidermal growth factor receptor or the p38 and ERK1/2 MAP kinase pathways, respectively [5]. Through FPRL1, LL-37 also activates endothelial cells, resulting in angiogenesis [20]. These activities account for the erythema, telangiectases and PMN infiltration of rosacea [3].

The polyanionic glycosaminoglycan heparin has been previously reported to inhibit LL-37 binding to lipopolysaccharide [21] and thereby reduce the antibacterial effects of LL-37 [22]. Heparin also neutralizes the toxicity of other cationic peptides, including eosinophil major basic protein and eosinophil cationic protein [23], [24]. This led us to hypothesize that the SAGEs could charge neutralize and reduce the biologic effects of exuberantly expressed LL-37 in humans with rosacea. The data from *in vitro* (Figure 4) and *in vivo* (Figures 5 and 7) experiments support this hypothesis. GM-1111 not only bound LL-37 (Figure 4A) and inhibited LL-37 when co-mixed with it prior to intradermal injection (Figure 5), but also when applied topically to the skin (Figure 7). Because LL-37 is also overexpressed in psoriatic skin, where it has been proposed to form complexes with human self-DNA to activate plasmacytoid dendritic cells and the processes that lead to lesions of psoriasis [5], [25], GM-1111 or other SAGEs might also prove therapeutically useful in other common inflammatory skin diseases.

In addition to blocking cathelicidin peptides, SAGEs are also potent inhibitors of RAGE with at least four of its disparate ligands (Table 1 and Figure 3). The pathogenic role of RAGE in skin diseases has only recently been explored. However, RAGE is already known to be centrally important in photo-ageing [9], the S100 calgranulins S100A7 and S100A17 are highly upregulated in psoriatic skin [26], and HMGB-1 has been recently reported to mediate cutaneous inflammation from topical croton oil [19]. Our finding that S100A8, the mouse homologue for human S100A12 [17], is increased in LL-37-injected skin (Figure 6) suggests that S100 calgranulins also may have a prominent role in the cutaneous inflammation seen in patients with rosacea.

RAGE is a promiscuous pattern recognition receptor from the immunoglobulin superfamily and plays a prominent role in magnifying inflammation [27], [28]. Once ligated, RAGE mediates post-receptor signaling including activation of nuclear factor-κB (NF-κB), leading to a profound inflammatory response [27], [28]. Through a prominent NF-κB-responsive consensus sequence in its promoter, RAGE activation also leads to even greater RAGE expression [27], [28]. Furthermore, RAGE interacts with the leukocyte Mac-1 integrin (CD11b/CD18) and p150,95 (CD11c/CD18) to facilitate leukocyte inflammatory cell recruitment [13]. Attraction of leukocytes to inflammation is additionally augmented by interaction of the RAGE ligands S100 calgranulins and HMGB-1 [13], [27]–[29].

Structurally, RAGE is comprised of three immunoglobulin-like regions: a distal “V” type domain, followed by two “C” type domains, a short transmembrane domain and a cytoplasmic tail required for signaling [27], [28]. The extracellular domain of RAGE has been used in detailed analyses of RAGE-ligand interactions [30] and the crystal structure of the receptor [31]. SAGEs likely inhibit RAGE-ligand interaction in part through interactions with the V domain, which consists of a large hydrophobic cavity rimed on its surface with basic amino acids to form a cationic center [30]. This is supported by a crystal structure for RAGE which reveals an elongated molecule with a large basic patch and a large hydrophobic patch, both highly conserved [31], which might offer attractive sites for binding of both acidic anionic and alkyl groups, respectively, of SAGEs. The dramatic differences among structurally similar SAGEs [6] and ligand specific structure-activity relationships strongly suggest that charge alone is only a small contributor to overall anti-inflammatory effects. Binding of polyanions such as SAGEs to this cationic region might be expected to not only inhibit binding of AGEs, which ligate RAGE at its V domain, but also, dependent upon the nature of substituent alkyl groups, to produce steric interference with the binding of other ligands such as S100/calgranulins and HMGB-1 which require adjacent C1 and/or C2 regions for full RAGE ligation [32].

A major issue with the use of sulfated polysaccarides such as SAGEs for topical treatment of skin diseases is their availability to the dermis. Literature on the bioavailability of sulfated polysaccharides across skin is conflicting, with some groups reporting little penetration but others suggesting transdermal absorption of heparin and unfractionated heparin as a result of their detergent properties [33]–[39]. In the most recently published work, fluorescent-labeled unfractionated heparin was found to adhere to keratinocytes in culture, and within 48 hours, almost half of topically applied labeled heparin was found to transcutaneously penetrate the skin of hairless rats mounted onto a Franz static diffusion cell [40]. Whereas SAGEs might permeate normal skin in humans less well than in mice, inflammation could serve to greatly increase SAGE penetration (Figure 9), as seen with other drugs used for serious skin diseases [41] and similar to the well-recognized enhancement of antibiotic penetration into cerebrospinal fluid in the presence of a disrupted blood-brain barrier in meningitis. Various techniques have been developed to enable transdermal delivery of low molecular weight (∼5,000 Da) heparin, including penetration enhancers [42], [43], liposomal formulation [43], [44], iontophoresis [45], [46] and low frequency ultrasound [45], [47], [48]. These strategies might also facilitate dermal penetration of similarly sized SAGEs to enable their effective topical use in skin disorders.

The chemistry and function of HA have been well explored in cutaneous biology, and bacterial and animal-derived HA continue to be an important component for the cosmetics, pharmaceutical and medical device industries. Our research suggests that simple chemical modifications of HA can generate novel anionic polysaccharides with broad anti-inflammatory activities. SAGEs have the additional advantage of being non-animal derived, thus circumventing the risks of adulteration and sourcing problems inherent in animal-derived sulfated polysaccharides [15]. Our observations suggest that SAGEs in topical application may prove to be a safe and useful treatment not only for rosacea, but also other inflammatory skin diseases.

## Materials and Methods

### Materials

Polyclonal goat anti-human RAGE (Cat# AF1145), recombinant HMGB-1, recombinant human P-selectin/Fc chimera, and recombinant human RAGE/Fc chimera were purchased from R&D Systems (Minneapolis, MN). Rabbit polyclonal IgG antibody to LL-37 (Cat# sc-50423) was from Santa Cruz Biotechnology (Santa Cruz, CA), and peroxidase-linked goat anti-rabbit polyclonal IgG (Cat# A0545) was from Sigma Aldrich (St. Louis, MO). Human S100b calgranulin was from Calbiochem (San Diego, CA). Alexa Fluor® 633 hydrazide was from Molecular Probes (Eugene, OR). The AGE product CML-BSA was from MBL International (Woburn, MA). Protein A, horse radish peroxidase (HRP)-conjugated rabbit polyclonal anti-goat IgG (Cat# 31402), carbonate-bicarbonate buffer and bovine serum albumin blocker (10x) were from Piercenet (Rockford, IL). Calcein AM, Dulbecco's modified Eagle's medium (DMEM), EpiLife medium, ethylenediamine tetraacetic acid (EDTA), fetal bovine serum (FBS), HEPES, non-essential amino acids, penicillin/streptomycin/L-glutamine solution, RPMI-1640 without L-glutamine, sodium bicarbonate, and tetramethyl benzidine chromogen (TMB) single solution chromogen were from Invitrogen (Carlsbad, CA). High-bind 96-well microplates were from Corning Life Sciences (Corning, NY), and heparin binding plates were from BD Biosciences (Bedford, MA). U937 monocytes and nHDF cells were purchased from American Type Culture Collection (Manassas, VA). nHEK cells were obtained from Invitrogen (Madison, WI). Antibodies for immunohistochemistry were sourced as identified in figure legends. All other chemicals not specified were from Sigma-Aldrich (St. Louis, MO). HA was obtained commercially from a recombinant *B. subtilis* expression system (Novozymes Biopolymers, Bagsvaerd, Denmark) or from streptococcal fermentation (LifeCore, Chaska, MN).

### Animal Care and Use

All animal protocols were approved by the University of Utah institutional animal care and use committee (IACUC). Approved protocols include 08-06002, 08-06005 and 08-11008.

### Characterization of SAGEs

A variety of SAGEs were prepared with four parameters varied: molecular weight, type of ether modification, substitution degree of ether modification, and degree of sulfation. These data are reported elsewhere [6]. Ether substitution degree (SD) of SAGE was determined by ^1^H NMR, and for GM-1111 was estimated to be SD  = 1, or ∼1 alkyl group per disaccharide unit. After sulfation, GM-1111 was dissolved in water, dialyzed, and lyophilized to give a white powder shown by ^1^H NMR to have a sulfation SD of 1.0–1.5. Fluorescent-labeled GM-1111 was synthesized by conjugation with Alexa Fluor 633 hydrazide.

Molecular weight was determined with gel permeation chromatography using a Waters 515 HPLC pump, Waters 410 differential refractometer, Waters 486 tunable absorbance detector, and Ultrahydrogel 250 or 1000 columns (7.8 mm i.d X 130 cm) (Milford, MA). Eluent was 200 mM phosphate buffer (pH 6.5): MeOH  = 80∶20 (v/v), and flow rate was 0.3 or 0.5 mL/min. The system was calibrated with standard HA samples from Dr. U. Wik (Pharmacia, Uppsala, Sweden). Average molecular weight of GM-1111 was ∼5,000–6,000 Da.

### Formulation of GM-1111 emollient

A 1% (w/w) emollient was prepared in Spectrum Transdermal Ointment (Lotioncrafters, Olga, WA), a commercially available proprietary ointment comprised of cetyl ricinoleate, carnuba wax, *D*-α-tocopheryl acetate, shea butter, caprylic triglyceride, lecithin and beeswax. GM-1111 (100 mg) was dissolved in 2.0 g of nanopure water, ointment was added to give 10.0 g, the mixture blended thoroughly and the emulsion processed through an ointment mill. Control emollient was made with 2 g water without active ingredient. Sodium hyaluronate emollient was prepared with 100 mg of 53 kDa HA (Novozymes, Bagsvaerd, Denmark) following an analogous protocol. Each formulation was stored at 4°C.

### Cell culture

U937 monocytes were grown in suspension at 37°C in 5% CO_2_-95% air in RPMI-1640 supplemented with 10% heat inactivated FBS, 2 mM L-glutamine, 1 mM sodium pyruvate, 0.1 mM MEM non-essential amino acids, 100 units/ml penicillin and 100 mg/ml streptomycin. nHDF cells were grown in DMEM supplemented with 10% FBS and penicillin-streptomycin. nHEK cells were grown in EpiLife medium supplemented with 0.06 mM Ca^2+^, 1% EpiLife defined growth supplement and penicillin-streptomycin. Experiments were performed with cells from passages 1-5.

### Factor XII activation assay

Five µl of pooled normal human plasma was incubated with 100 µl of GM-1111 ranging from 0.1-1000 µg/ml in 0.05M HEPES containing 0.05% TritonX-100 for 5 min at 25°C. Amidolytic activity was determined with 0.5 mM H-D-CHT-Gly-Arg-*p*NA by following the change of optical density (OD) for 30 min at 405 nm [16]. The OD obtained at 30 min was plotted against the concentrations of the activator.

### Cell surface binding assays

The effect of GM-1111 on binding of U937 monocytes to P-selectin or RAGE was studied using calcein labeled cells incubated in micro plates coated with P-selectin-Fc or RAGE-Fc chimeras. These methods have been reported in detail [10].

### Inhibition of human leukocyte elastase (HLE)

Inhibition of HLE by SAGEs was determined using the specific chromogenic substrate suc-Ala-Ala-Val-*p*NA, according to methods previously described [10].

### Solid phase binding assays

Three types of ELISAs were performed: one to study the binding of vascular adhesion molecules and LL-37 to GM-1111, and another to study binding between RAGE and its ligands, including CML-BSA, HMGB-1 and S100b. A competitive ELISA was also performed to study the ability of GM-1111 to inhibit/compete RAGE binding to its ligands. These ELISAs have been reported in detail [10].

### *In vitro* cytotoxicity and anticoagulant assays

nHDF cells were seeded (4,000/100 µl medium) in each well of 96-well flat-bottomed microplates, and incubated at 37°C in 5% CO_2_ for 12 hours. Medium was changed with complete medium containing GM-1111 at final concentrations of 10 to 10^6^ ng/ml to each well. At 48 hours, 20 µl MTS (CellTiter96® Aqueous One assay; Promega, Madison, WI) was added to each well, and cells were further incubated for 2 h. Absorbance of the samples was measured at 490 nm using a 96-well plate reader to determine cytotoxicity. Cytotoxicity was assessed similarly in nHEK cells. Automated amidolytic assays for anti-Xa and anti-IIa activity were performed by BioCascade, Arlington, WI.

### Skin irritation tests in mice

SAGEs were tested *in vivo* to assess dermal irritation potential. GM-1111 was prepared at 0.1, 1 and 10 mg/ml. Formic acid (10%) and PBS were used as positive and negative control, respectively. Balb/c mice (n = 6 per group), free from skin irritation, trauma, or adverse clinical signs prior to study, were randomized and grouped for test conditions. Backs of animals were clipped free of fur. Each mouse received four parallel epidermal abrasions with a sterile needle at the bottom area of the test site, while the upper area of the test site remained intact. Under isofluroane anesthesia, two 0.5-ml samples of the test solution were applied to the entire test site under a double gauze layer to an area of skin approximately 2.5 cm^2^. Patches were backed with plastic, covered with a non reactive tape and the test site wrapped with a bandage. After 24 h exposure to the agent, the bandage and soaked test gauze were removed and test sites were wiped with tap water to remove remaining test compound. At 24 and 72 h after application, test sites were examined for dermal reactions in accordance with the FHSA- recommended Draize scoring criteria [14]. Primary Irritation Index (P.I.I.) of test article was calculated following test completion. A material producing a P.I.I. score of greater than or equal to 5.00 would be considered positive and be classed as a primary irritant to skin.

### Peptide synthesis

LL-37 was prepared by the core peptide facility at the University of Utah, containing the amino acid sequence: LLGDFFRKSKEKIGKEFKRIVQRIKDFLRNLVPRTES. Synthetic peptides were purified to >95% by HPCL and sequence was confirmed by mass spectrometry.

### LL-37-mediated skin inflammation models

To study the effect of SAGE in rosacea, we used a previously reported disease model produced by intradermal injection of LL-37 [3]. Balb/c mice were shaved prior to study to expose skin on the back. Twenty-four hours later, 40 µl of vehicle (nanopure water), LL-37 (320 µM), SAGE (1,280 µM), or LL-37+ SAGE mix (peptide + 4x molar concentration of SAGE) was injected intradermally into the shaved skin using a 0.5 ml syringe and 31-gauge insulin needle in a manner to raise an intact dermal bleb, identifying that administration was at the lower epidermis or dermis. To standardize results, all intradermal injections were performed by a single investigator with experience performing intradermal injections on the human volar forearm with needle bevel facing upward. SAGE and LL-37 were mixed together in PBS and allowed to incubate 15 min at room temperature before injection. Injections were repeated every 12 h thereafter. Forty-eight hours after the initial injection (four injections in total), animals were anesthetized with isofluorane. Injected skin was photographed to record severity of erythema and edema. Mice were euthanized with CO_2_, and injected skin was excised for hematoxylin and eosin staining and immunochemistry. From the center of excised skin, a 6 mm punch biopsy was obtained, weighed, snap frozen in liquid N_2_ and stored at -80 C for measurement of tissue MPO.

To study topical SAGE in this model, mice were injected with LL-37 as described above. GM-1111 was then applied topically as a 1% concentration in 50 µl of emollient, either immediately after the first LL-37 injection or 12 h thereafter, with repeated application every 12 h after each LL-37 injection. After application, emollient was massaged into the affected area of skin with a gloved finger rubbed 32 consecutive times in a counter-clockwise direction. Control animals were treated similarly with emollient alone. To standardize treatment, all topical medication applications were performed by a single investigator after the individual performing intradermal injections had finished the injections and left the laboratory area. After four LL-37 intradermal injections, mice were photographed and biopsied as above.

### Croton oil inflammation model

As another model of PMN-mediated skin inflammation, we employed croton oil [18]. Croton oil (0.8% solution in acetone) was painted (10 µl each side) on one ear of Balb/c mice, with the other ear as a control. Fifteen minutes later, GM-1111 was then dosed topically as a 1% concentration in 50 µl of emollient applied to each side of the croton-oil treated ear. After application, emollient was massaged into each side of the ear with a gloved finger rubbed 32 consecutive times in a counter-clockwise direction. Control animals were treated in a similar fashion with emollient alone. To standardize treatment, all topical medication applications were performed by a single investigator after the individual performing croton oil applications had left the laboratory area. After 4, 8 and 24 h, ear thickness was measured near the top of the ear distal to the cartilaginous ridges. Change in ear thickness from control was taken as an edema index. Following 24 h measurements, mice were euthanized with CO_2_ and 6 mm ear punch biopsies taken, weighed, frozen and stored at −80°C for determination of MPO activity. A single investigator performed all measurements and biopsies in order to standardize the procedure. Remaining ears were removed, embedded and frozen for hematoxylin and eosin stains and immunohistochemistry.

### MPO assay

Tissue MPO activity was measured using the method of Suzuki et al. [49] modified by Young et al. [50]. Biopsies were placed in 0.75 ml of 80 mM PBS (pH 5.4) containing 0.5% hexadecyltrimethyl-ammonium bromide (HTAB). Each sample was homogenized for 45 s at 4°C with a Tissue Tearor Homogenizer (Model 985-370; Biospec Products, Bartlesville, OK). Homogenate was transferred to a microcentrifuge tube with an additional 0.75 ml HTAB in PBS. The 1.5 ml sample was centrifuged at 12,000×*g* for 15 min at 4°C. Triplicate 30 µl samples of the resulting supernatant were added to 96-well microtiter plates. For MPO assay, 200 µl of a mixture containing 100 µl of 80 mM PBS (pH 5.4), 85 µl of 0.22 M PBS (pH 5.4), and 15 µl of 0.017% hydrogen peroxide were added to each well. To this, 20 µl of 18.4 mM tetramethylbenzidine HCl in 8% aqueous dimethylformamide (DMF) was added to start the reaction. Microtiter plates were incubated at 37°C for 3 min, and placed on ice. The reaction was stopped with the addition of 30 µl of 1.46 M sodium acetate. MPO activity was assessed at 630 nm and expressed as optical density (OD)/biopsy.

### Immunohistochemistry

LL-37, PBS and LL-37 and SAGE-injected mouse skin was stained with antibody to mouse Gr-1 to examine neutrophil infiltration, and also for S100A8 calgranulin, the major RAGE-binding calgranulin present in murine PMNs [17]. Slides were deparaffinized and hydrated through Citrisolv and graded ethanol washes. Endogenous peroxidase activity was blocked with 1% H_2_O_2_ in PBS with 0.1% Tween-20 (PBST) for 20 min. Antigen retrieval was performed by microwaving in 1% antigen unmasking solution (Vector Laboratories) for 20 min followed by incubation at room temperature for 30 min. Immunostaining was performed using the Vectastain Elite ABC peroxidase kit (Vector Laboratories). Briefly, non-specific antibody binding was minimized by incubating sections for 90 min in diluted normal blocking serum. Sections were incubated overnight at 4°C in a humidified chamber with primary goat anti-mouse calgranulin A antibodies (Santa Cruz #sc-8113) at a 1∶200 dilution and rat anti-mouse Gr-1 (R&D Systems #RB6-8C5) at a 1∶500 dilution. Following overnight incubation, slides were washed in PBST for 9 min, incubated 2 h with biotinylated secondary antibody diluted to 5 µg/ml in PBST, followed by Vectastain Elite ABC Reagent (Vector) diluted in PBST for 30 min. Between incubations, sections were washed for 12 min in PBST. Immunoreactivity was detected by incubating with the DAB peroxidase substrate kit (Vector Laboratories) for 1–2 min. Sections were then washed in nanopure water and counterstained with hematoxylin before dehydration and mounting with coverslips.

### Statistical Analysis

All experiments were performed in triplicate or quadruplicate for *in vitro* tests. Results are expressed as means ± standard error of the mean (SEM). Significant differences between samples were calculated by comparison of means using the Aspin−Welch test. In experiments with multiple groups or treatments, a one-way analysis of variance (ANOVA) followed by Student-Newman-Keuls *post hoc* test was used to analyze for group differences. Significance was declared at P<0.05.
